# Coherent Excitation and Control of Plasmons on Gold Using Two-Dimensional Transition Metal Dichalcogenides

**DOI:** 10.1021/acsphotonics.0c01795

**Published:** 2021-05-26

**Authors:** Jan Vogelsang, Lukas Wittenbecher, Deng Pan, Jiawei Sun, Sara Mikaelsson, Cord L. Arnold, Anne L’Huillier, Hongxing Xu, Anders Mikkelsen

**Affiliations:** † Department of Physics, Lund University, Box 118, 22100 Lund, Sweden; ‡ Nano Lund, Lund University, Box 118, 22100 Lund, Sweden; § School of Physics and Technology and Key Laboratory of Artificial Micro- and Nano-structures of Ministry of Education, Wuhan University, Wuhan 430072, China; ∥ Institute for Advanced Studies, Wuhan University, Wuhan 430072, China

**Keywords:** surface plasmon polaritons, ultrafast plasmonics, transition metal dichalcogenides, metal semiconductor hybrid systems, time-resolved photoemission electron microscopy

## Abstract

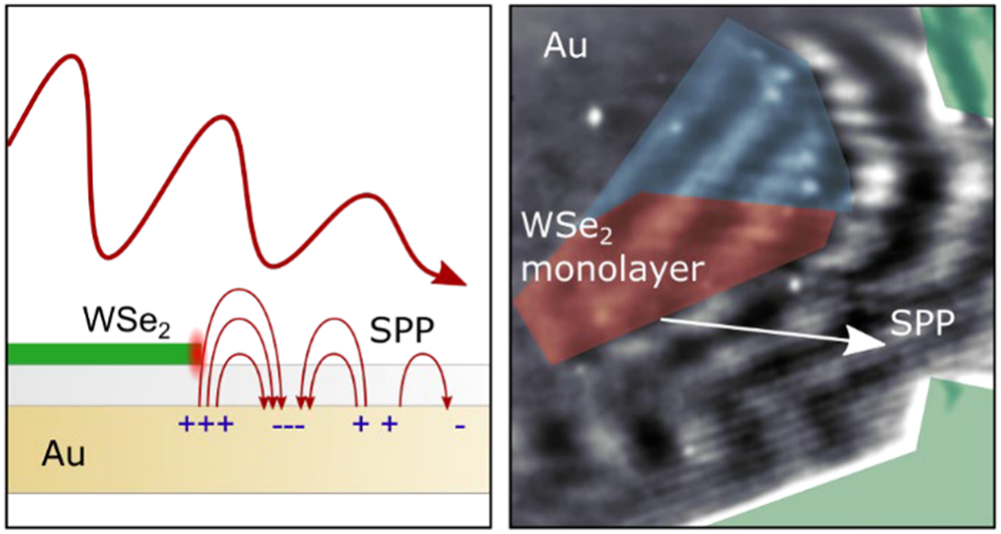

The hybrid combination of two-dimensional (2D) transition metal dichalcogenides (TMDs) and plasmonic materials open up novel means of (ultrafast) optoelectronic applications and manipulation of nanoscale light–matter interaction. However, control of the plasmonic excitations by TMDs themselves has not been investigated. Here, we show that the ultrathin 2D WSe_2_ crystallites permit nanoscale spatially controlled coherent excitation of surface plasmon polaritons (SPPs) on smooth Au films. The resulting complex plasmonic interference patterns are recorded with nanoscale resolution in a photoemission electron microscope. Modeling shows good agreement with experiments and further indicates how SPPs can be tailored with high spatiotemporal precision using the shape of the 2D TMDs with thicknesses down to single molecular layers. We demonstrate the use of WSe_2_ nanocrystals as 2D optical elements for exploring the ultrafast dynamics of SPPs. Using few-femtosecond laser pulse pairs we excite an SPP at the boundary of a WSe_2_ crystal and then have a WSe_2_ monolayer wedge act as a delay line inducing a spatially varying phase difference down to the attosecond time range. The observed effects are a natural yet unexplored consequence of high dielectric functional values of TMDs in the visible range that should be considered when designing metal–TMD hybrid devices. As the 2D TMD crystals are stable in air, can be defect free, can be synthesized in many shapes, and are reliably positioned on metal surfaces, using them to excite and steer SPPs adds an interesting alternative in designing hybrid structures for plasmonic control.

## Introduction

Two-dimensional crystals of transition metal dichalcogenides (TMDs) have been extensively studied in recent years due to their many potential applications in optoelectronics.^1−3^ TMDs are characterized by a distinct layered structure, making the fabrication of 2D crystals similar to graphene possible. Quantum confinement and a reduced dielectric screening change the carrier dynamics and the correlated electron behavior, leading to the presence of interesting exciton phenomena on an ultrafast time scale.^4^ To amplify and control the excitations in TMDs spectrally, as well as in time and space, surface plasmon polaritons (SPPs) have recently gained significant interest.^5−8^ To induce SPPs by light, the spatial symmetry of the surface has to be broken due to momentum conservation. This has usually been accomplished by synthesizing nanocrystallites with naturally confined borders like silver rods or gold flakes^7^ or by manufacturing tens of nanometers deep/high holes, ridges, protrusions, or other morphological variations in metal films.^5,9^ By combining plasmonic materials with single molecular layers, it has been found that SPPs can be incoherently excited by excitons in 2D materials^10^ or by specifically prepared tunnel junctions.^11^ However, the ability to form atomic scale precise boundaries and layer thicknesses in TMD thin films has not been used for coherent SPP excitation and manipulation, despite the high values of the dielectric functions,^12^ yielding refractive indices between 4 and 5 in the visible range. Nonetheless, this would be highly desirable due to the widespread use of propagating SPPs at metal–dielectric interfaces, which with perfect crystalline systems could lead to coherent control with subfemtosecond temporal precision and low losses in realistically defect free structures.

Here, we propose and demonstrate spatially precise SPP excitation as well as subfemtosecond temporal control on a flat metal surface by depositing 2D TMD crystals. The SPP spatiotemporal behavior is governed by the molecular well-defined thicknesses and edge shapes of the TMDs. With no demands of lateral structuring of the metal substrate and the use of exfoliated TMDs, a metal–dielectric hybrid system with a low defect density and morphological perfection can be achieved which should enable low losses of the excited SPPs.

After excitation, SPPs are altered when they pass through the 2D material^13−15^ compared to propagation at a metal–vacuum interface due to the different dielectric functions. This well-known observation for dielectrics on top of metal films^16,17^ can be applied to, for example, construct waveguides.^18^ SPP manipulation has also been achieved for self-assembled monolayers of organic molecules,^16,19^ which are however unstable in ambient air.^20^ Here, using the 2D material with a thickness of a single molecular layer, a small and precise variation of the SPP propagation is possible, while the material remains stable in ambient air. As a result, the readily achievable control of the 2D material’s lateral dimension within a few tenths of nanometers allows the TMDs to act as surface optical elements to control SPP dynamics with a precision corresponding to a few attoseconds. Further, given that SPP manipulation using thick dielectric films has been understood in a mean field approximation, it will be interesting to explore when and if this holds true in 2D TMDs that reach the atomic scale. The study of femto- and attosecond electron dynamics in nanostructures is a thriving topic in itself, to which the present system could make significant contributions^21,22^ due to its variable, but well-defined, nature.

To directly observe the spatiotemporal SPP manipulation using two-dimensional WSe_2_, we use interferometric time-resolved photoemission electron microscopy (ITR-PEEM). This technique has been highly successful in studies of dynamics with extreme temporal and spatial resolution.^23−28^ ITR-PEEM has, among very few other techniques,^29^ the important advantage of avoiding the detrimental effect of electron pulse broadening on the temporal resolution of the ultrafast electron microscopy experiment. It combines the few-nanometer spatial resolution of electron microscopy with the femtosecond resolution of interferometric optical techniques^30,31^ with the prospect of expanding this to the attosecond regime.^32,33^

In the present work, we demonstrate, investigate, and use the excitation of SPPs at a monocrystalline gold vacuum interface from the edges of thin WSe_2_ crystals. Although such a crystal can be 3 orders of magnitude thinner than the SPP wavelength, we find that already a monolayer is sufficient to launch SPP waves by light scattering at the edges. We demonstrate that the complex interference patterns^34,35^ observed between the launched SPPs and the incident laser field can be simulated by simple assumptions about the scattered waves and knowledge of the incoming exciting light. We then use the SPPs launched by the sharp edge of a WSe_2_ flake using few-femtosecond laser pulses, to observe interferometrically the temporal behavior of the SPPs as they are dispersed by a wedge-shaped prism-like WSe_2_ crystal. For a detailed characterization of the sample and the optical setup, please refer to the Supporting Information (SI).

## Results and Discussion

### Nanoscale Spatial Control of SPP Excitation

**Figure 1:**
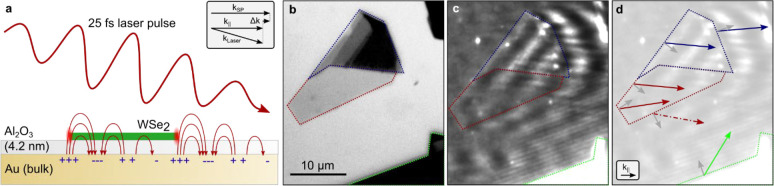
Surface plasmon polaritons launched on Au from the edges of WSe_2_ crystals. a, Schematic drawing of the sample and the SPP’s excitation mechanism. b, Optical image of a WSe_2_ crystal (in the center) and of several other thick crystals (the edges). Different layer thicknesses of the WSe_2_ crystals can be identified by the gray levels of the images. The monolayer region of the central WSe_2_ crystal is marked with a red dotted line, few layers in blue, and thicker multilayers in light green. c, Photoemission electron microscope image taken when illuminating the sample in (b) with a short laser pulse at a central wavelength of 715 nm. The wave patterns with different orientations and periodicities show the SPP launched on the surface. The laser is incident from the left at an angle of 65° to the surface normal. d, Propagation directions of SPPs excited at selected edges of the WSe_2_ crystals indicated by red, blue, and green arrows, calculated using Snell’s law. One of the SPPs launched by a monolayer of WSe_2_ is indicated by a dashed-dotted arrow. The *k*-vectors of the interference pattern (which are actually observable) are indicated by shorter light gray arrows. The laser’s in-plane wave vector *k*_∥_ is indicated by the arrow on the bottom left.

The basic SPP excitation by WSe_2_ in our experiment is illustrated in Figure 1a. A short laser pulse illuminates the sample consisting of a monolayer and few-layer crystals of WSe_2_ on a monocrystalline gold film coated with a 4.2 nm-thin spacer layer of Al_2_O_3_ (see Materials and Methods) at an angle of 65° to the surface normal. When the illumination is chosen such that the illuminated region is partially covered by the WSe_2_ crystal, the spatial symmetry is broken at the edges of the WSe_2_ crystal. The light field passing through the monolayer will have a different relative phase than light reaching the Au surface directly. In that sense, the edge of the material with its high refractive index and atomically sharp border locally broadens the momentum spectrum available for SPP excitation due to the introduced phase jump.^36,37^ This compensates the difference Δ*k* in the laser’s in-plane wave vector *k*_∥_ and the SPP wave vector *k*_*SP*_ and leads to the SPP launch at the Au–Al_2_O_3_–vacuum interface. The SPP can propagate in both directions: away from and through the WSe_2_ crystal (Figure 1a, backscattered SPPs are not shown for clarity). The relative excitation strengths depend on the laser’s angle of incidence and the thickness of the WSe_2_ crystal. Regions of the WSe_2_ crystal with multiple layers and a large monolayer region in the center can be identified on an optical microscope image (Figure 1b). Additionally, the monolayer nature of the crystal has been verified by atomic force microscopy (see SI).

The PEEM image of the crystal (Figure 1c) is the result of the photoelectron emission by a 25 fs fwhm light pulse with a 30 nm broad spectrum centered around 715 nm (the laser spectrum supporting a pulse duration of 6 fs was spectrally cut to increase SPP visibility) illuminating the sample with an in-plane wave vector pointing to the right under an angle of 65° to the surface normal. Since the emitted photoelectrons displayed in the PEEM image result from three to four photon multiphoton excitation (see the SI), the contrast will be proportional to the coherent sum of the light field and SPPs launched on the surface raised to the power of six to eight (see Materials and Methods and the SI for more details on the imaging mechanism). The leading part of the laser pulse excites SPPs that can interfere with the trailing part of the pulse on the surface. This results in a pattern of destructive and constructive interference on the sample surface,^25^ demonstrating the efficient launching of SPPs. A complex periodic modulation pattern of the near-field with different periodicities and angles can be observed resulting from SPPs launched from different edges of the WSe_2_ crystals and their interference with the laser field. Further, a decreasing interference amplitude for progressively thinner crystals is seen in Figure 1c. Remarkably, even the height of a monolayer of WSe_2_ on the Au substrate (marked by dotted red lines) is sufficient to launch an SPP to the bottom right direction of the PEEM image, resulting in the visible interference pattern (see also the red dashed-dotted arrow in Figure 1d). This SPP can even be observed in the presence of other SPPs launched by several layers of the WSe_2_ crystal (marked by dotted blue and green lines in Figure 1c).

**Figure 2:**
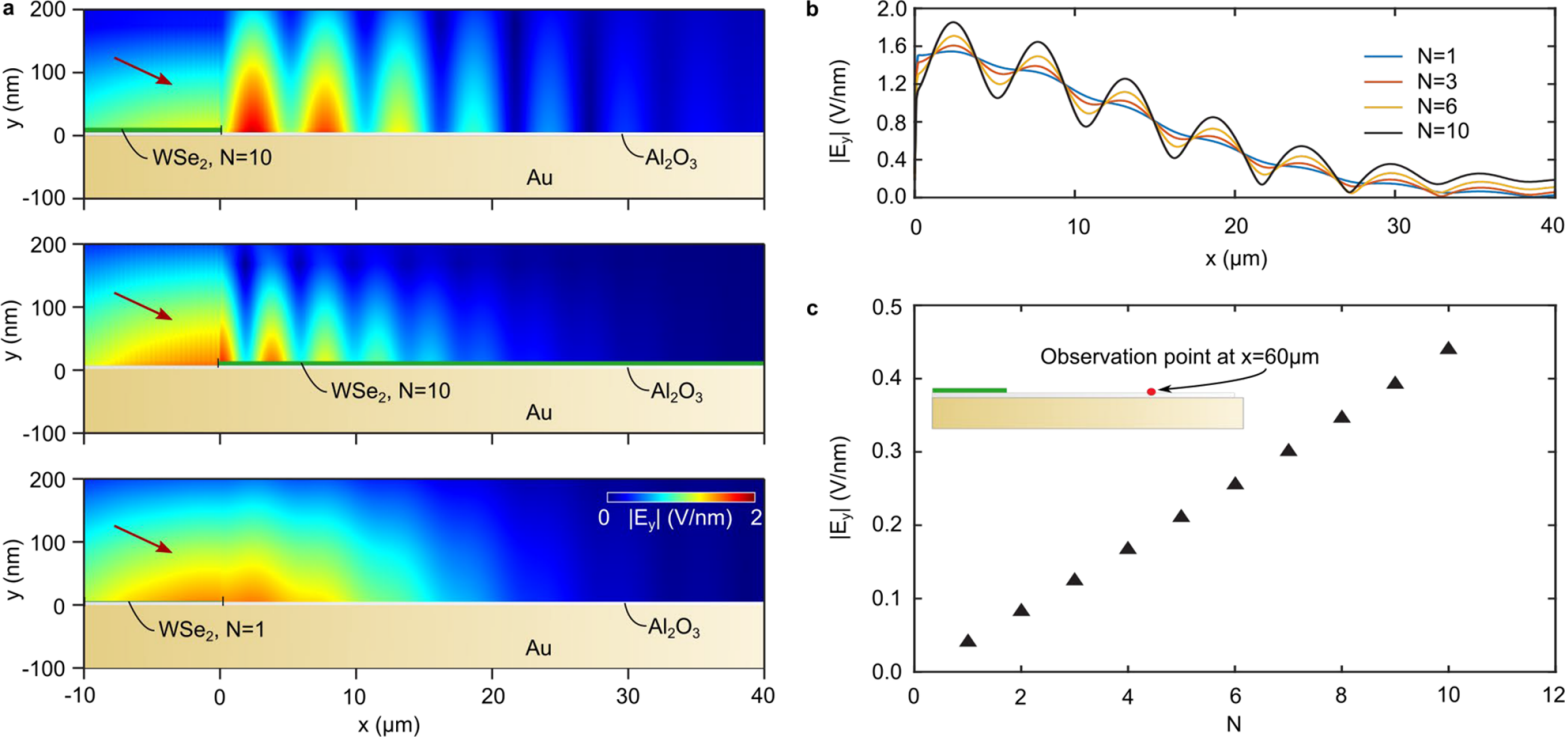
Simulations of the effective SPP excitation by WSe_2_ edge scattering. a, Simulated field distributions of the SPP generation. The SPPs excited near the WSe_2_ edge (at origin *x*, *y* = 0) can propagate into the bare region (top and bottom) or the region covered by WSe_2_ (middle). Excitation is possible for both a large number of layers (*N* = 10) and just a monolayer (*N* = 1, bottom). The laser angle of incidence is indicated by a red arrow. b, Field distribution on the Al_2_O_3_ surface for the configuration in the bottom panel in (a) with increasing N. c, Excited SPP field strengths for different *N* values. To reveal the SPP excitation efficiency compared to the incident field of 1 V/nm, the electric field is recorded at a point far away from the incident Gaussian beam (see inset), and the ohmic loss is ignored.

The visible SPP generation by a few layers of WSe_2_ is confirmed by simulations, as shown in Figure 2. Here, we use a finite element method to simulate the near field distribution with a laser beam illuminating a 1D representation of the sample system studied experimentally (see Materials and Methods). This turns out to adequately describe the SPP generation process observed in the experiment. As shown in the top and middle panels in Figure 2a, for a WSe_2_ crystal with 10 molecular layers (*N* = 10), SPPs are effectively excited as explained above, and clear fringes resulting from the interference between the SPPs and the incident fields are observed. The SPPs can propagate into both the bare region (top) and the region covered by WSe_2_ (middle), with the relative intensity depending on the in-plane component of the incident photon momentum. Consistent with the experiment, a monolayer of WeS_2_ is sufficient to excite the SPPs, as shown in the bottom panel of Figure 2a. We note that the decay of the fringe visibility in our simulations is mainly due to the finite waist of the incident Gaussian beam used in the simulation.

The dependence of the SPP excitation efficiency on the number of WSe_2_ layers is shown in Figure 2b. Here, the field distribution at the surface is recorded when the SPPs propagate into the bare region, such as the configuration in the top and bottom panel of Figure 2a. For small *N*, the fringe visibility is reduced because of a comparably weak SPP field. To estimate the SPP excitation efficiency from the simulation, we suppress the ohmic loss of the Au and observe the field intensity at a distant point from the WSe_2_ edge. Here, the incident field is negligible and the SPP field can be extracted for different *N* values (Figure 2c). The dependence is in qualitative agreement with the attenuation seen experimentally in Figure 1c: SPPs scattered in forward direction are observed with a higher interference amplitude for increasing layer thicknesses from monolayers to few- and multilayers. Still, also at a monolayer thickness of less than a nanometer, SPPs are excited with an efficiency of a few percent. This is much thinner than in the usual excitation geometry of plasmonic materials which employs metallic edges at least tens of nanometers high.^9,25,30,36,37^ SPP excitation by step edges in the plasmonic material has not been observed experimentally for heights below the tens of nanometer range, and theoretical modeling indicates that such SPP excitation efficiency falls close to zero below ∼20 nm.^36,37^ As explained above, the excitation mechanism is slightly different here: We use a dielectric with a sharp boundary and high refractive index to introduce a phase jump on the Au surface as explained above. Despite the thin dielectric layer, this leads to visible SPP excitation. Additional simulations (included in the SI) show that the presence of the WSe_2_ is indeed central as a monolayer step of Al_2_O_3_ will not lead to any significant SPP excitation. Instead the main role of the Al_2_O_3_ is to act as an atomic precise spacer and electrical insulator of the semiconducting WSe_2_ from the metallic Au, which opens it up for future device applications and interface coupling.

**Figure 3:**
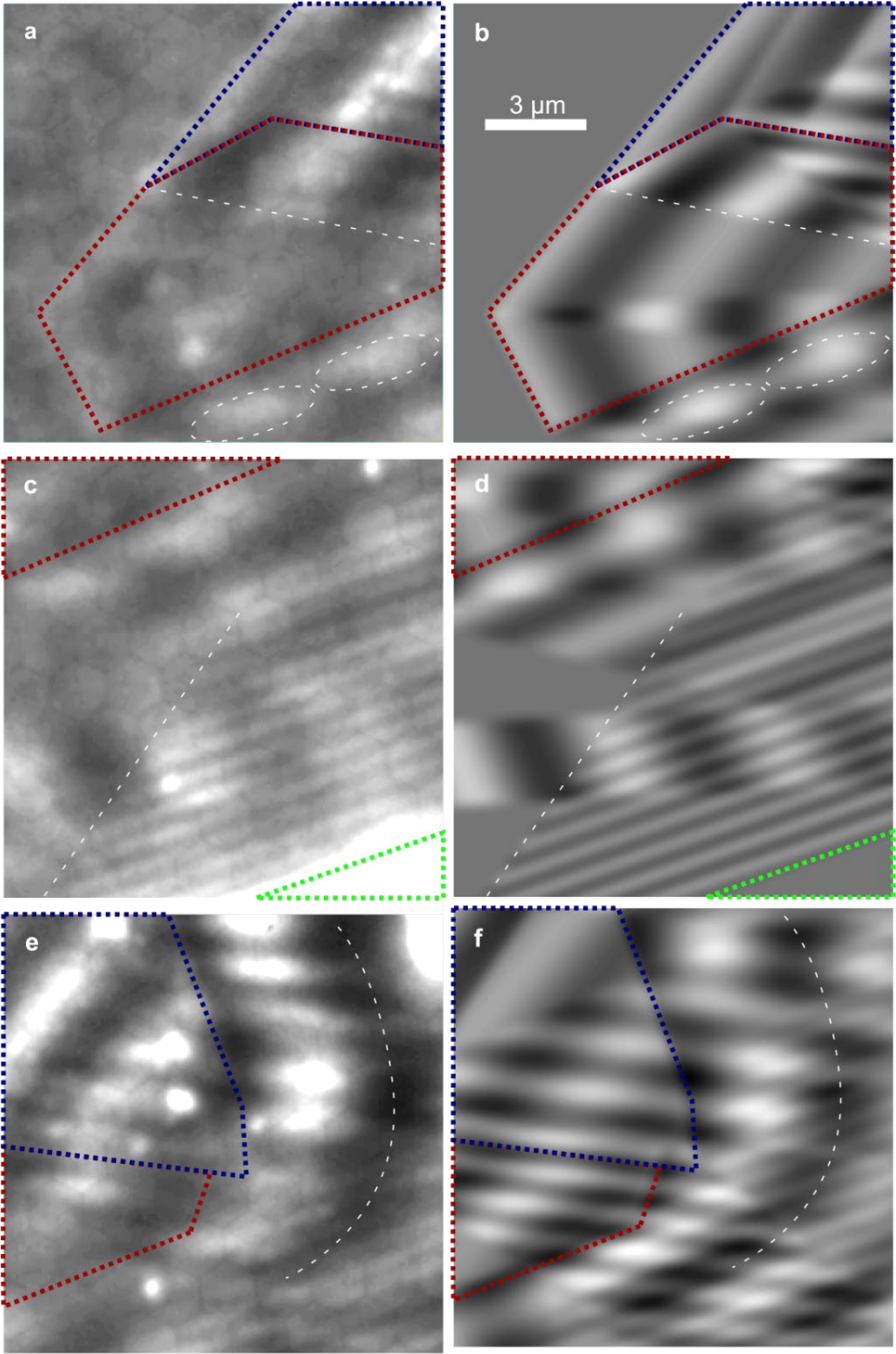
Comparison of experimental results (a, c, e) with numerical calculations (b, d, f) of SPP patterns. a, c, and e, Images that are magnifications of parts of Figure 1c. Edges of monolayer (red), few-layer (blue), and bulk (light green) WSe_2_ crystal parts are marked with dotted lines. b, d, and f, Numerical simulation of the SPP interference patterns in the respective regions selected in (a), (c), and (e). The different wavelengths of the interference patterns, propagation directions, and appearance of high intensity regions due to interference are nicely reproduced. White dashed lines are drawn to help the comparison to the experimental images.

With the principle of the SPP excitation established for the 1D case, we can go on to reproduce the full 2D images observed in the experiment. In Figure 3a,c,e, we show selected magnified regions from the image in Figure 1c and simulate these patterns as shown in Figure 3b,d,f. We model the experimentally observed interference patterns using the positions of the WSe_2_ crystal edges determined from the optical image (Figure 1b). We analytically calculate the SPP propagation direction and wavelength after laser excitation^34^ and numerically overlay the SPP and laser fields to get the interfering electric fields excited at the different edges (see Materials and Methods). Finally, we use these fields to calculate the electron yield for each position in a third order nonlinear emission process. The resulting images can be compared to the experimental images and are plotted next to them (Figure 3b,d,f). Excellent agreement can be found in particular for the local maxima appearing due to interference of SPPs excited from different edges. This proves that although the interference patterns look complicated at first sight, they can be well predicted with a rather simple geometric model, and in turn the design of SPP generators for, e.g., the purpose of optical signal processing is possible.^38^

Another interesting feature is the SPP generated at the thicker part of the WSe_2_ crystal (light green in Figure 3c,d), which travels to the top right in the image, leading to a high-frequency modulation with a wave vector of the interference pointing to the top left. This interference pattern is very similar to normal-incidence illumination since the in-plane wave vector of the laser in the SPP propagation direction is small. In general, the areas where the interference patterns from a particular edge are visible end at a sharp boundary (for example, indicated by the white dashed line in Figure 3c,d). This boundary very nicely illustrates the actual propagation direction of the SPP, since the interference pattern only occurs where an SPP field was excited.

### Temporal Investigations of the 2D TMD Induced SPPs

While we have demonstrated the possibility to spatially control the SPPs by WSe_2_ crystal edges, a highly interesting further area to study is the temporal regime and the possibilities of control using nanostructured optical components on the surface. In Figure 2a (top and middle panels), we observe a clear difference between the period of the fringes in the bare region and those in the WSe_2_ region, which originate from the different wavelengths of the SPPs in these two regions, considering that the illumination conditions remain the same. Thus, the 2D WSe_2_ provides a flexible way to influence the SPP propagation by precisely controlling the flake extension and layer number *N*. To make a first exploration of these dynamics, we locate two WSe_2_ crystals in a specific geometry and use the propagation of SPPs excited at the edge of one WSe_2_ crystal to explore how they propagate through a second wedge shaped 2D WSe_2_ crystal. We make use of the full spectrum of the laser system to deliver pulses with a duration of only ∼7 fs (full width half-maximum, see the SI). In order to extract quantitative information, we compare the propagation of SPPs in different regions using laser pulse pairs with variable delay Δ*t* generated in a Mach–Zehnder type interferometer (see Materials and Methods).

**Figure 4:**
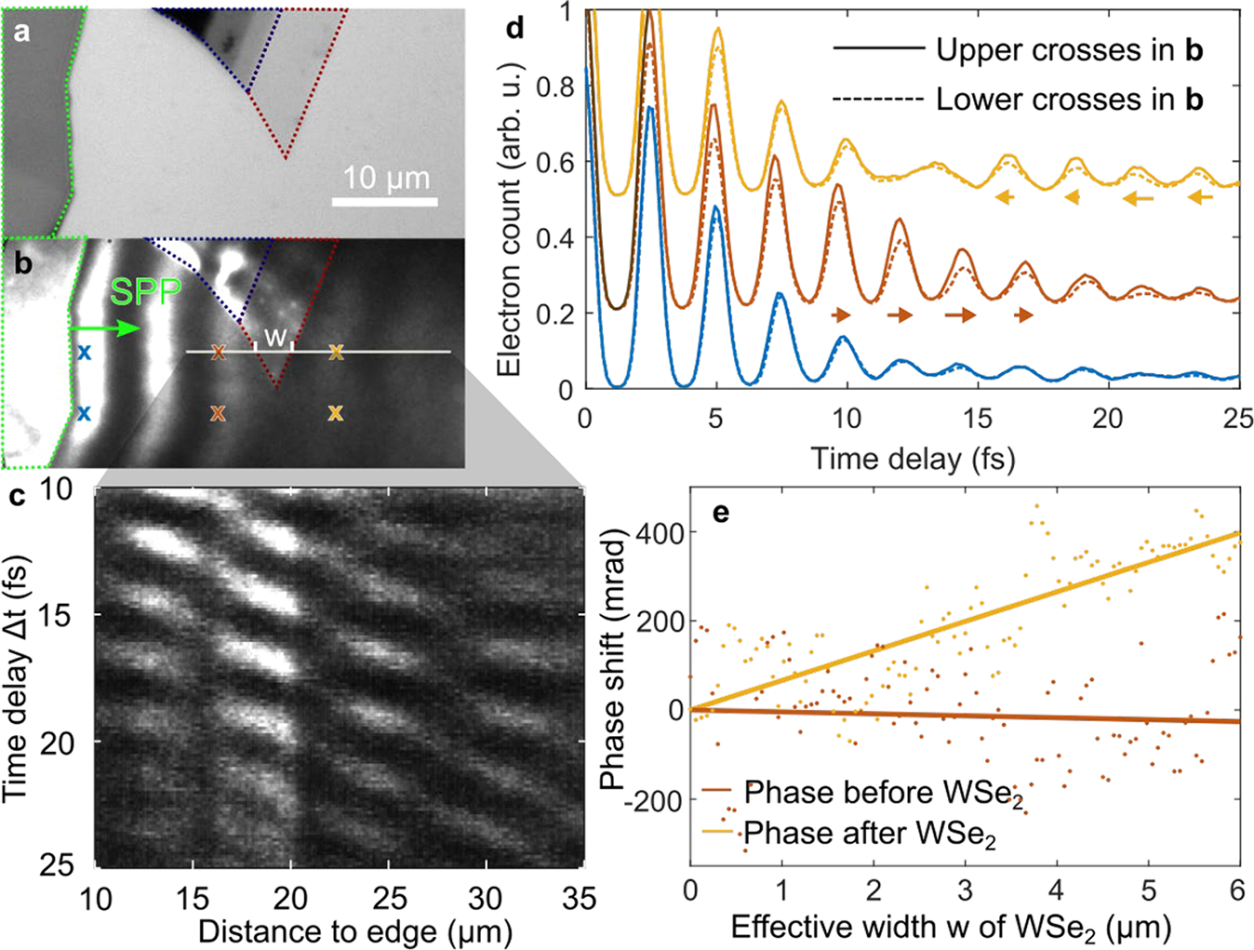
Delay experiments of an SPP by propagation through a WSe_2_ monolayer. a, Optical image of the sample. The monolayer region in the top middle is marked with a red dotted line, the few-layer region next to it in blue, and a thicker multilayer region on the left in light green. b, PEEM image of the sample when illuminated with ultrashort laser pulses. c, Measured 2D space time diagram of the electron count rate along the solid line in (b). The vertical modulation (in the time delay direction) results from the interference of the second laser pulse with the SPP launched by the first pulse at the WSe_2_ edge on the left in (b), which explains why the phase of the vertical modulation shifts when moving to the right. The horizontal modulation showing four oscillation periods in (c) is a remainder of the static interference pattern which is more pronounced close to the bulk edge. d, Electron counts at six different positions (marked with crosses in (b)) when the time delay of the pulse pair is changed. Vertically aligned positions are compared. The propagating SPP becomes visible by an increased peak height around time delays of 12 fs (red) and 20 fs (yellow) compared to the start position (blue). Its position depends on the distance to the excitation edge. While the SPP in the red curves shows a later arrival on the dashed line due to delayed SPP excitation, the shift changes sign after the upper part of the SPP has passed through the WSe_2_ monolayer (yellow). e, Relative phase of the temporal SPP oscillation before (red) and after (yellow) it has propagated through different widths *w* of the WSe_2_ monolayer. The phase was systematically extracted on horizontal lines for different material widths *w*. Linear fits approximate the data in order to extract the impact of the material on the phase velocity of the SPP oscillation. We find a phase shift of 70 mrad/μm. Note that the phase was extracted for every line on the camera, although the signals recorded on neighboring pixels were not independent due to resolution of the PEEM on the screen being lower than the camera resolution.

The first laser pulse excites a broadband and ultrashort SPP at a bulk edge of a WSe_2_ crystal shown in the optical image in Figure 4a on the left. The SPP propagates to the right, and part of it passes a piece of monolayer WSe_2_ (Figure 4b). At temporal delays Δ*t* > 30 fs, the second laser pulse is effectively independent of the first one, and a static interference pattern not depending on Δ*t* is recorded (as before). In contrast to this, for Δ*t* < 30 fs, temporal dynamics can be observed. While the first 10 fs are dominated by the interference of the laser pulses, the range between 10 and 30 fs gives a clear insight into the SPP dynamics on the surface as the SPP propagates through WSe_2_. The short laser pulse duration of only 7 fs makes this separation of laser and plasmon dynamics possible before the SPPs have decayed. This is shown in a spatiotemporal map in Figure 4c. It represents the electron count rate along the solid line in Figure 4b for different time delays Δ*t*. By choosing the vertical position of the line, a width *w* of the WSe_2_ monolayer piece of 4 μm is selected here. The propagation of the SPP through the monolayer induces an additional delay to its phase. This shift is visualized in Figure 4d by directly comparing the electron count rates for different time delays at the six vertically aligned positions marked in Figure 4b. From these single point delay scans it can be seen that the profiles recorded at the launch of the wave are in phase at all times (blue). When the SPP time structure is explored further away from its starting point, clear phase differences are observed (red, yellow). For the positions in the middle, the lower SPP appears with a small delay of −107 mrad (see the SI) on the reference line due to its excitation further on the left. This behavior changes after the upper SPP on the solid line has propagated through the WSe_2_ monolayer. Now, we find a phase delay of 170 mrad. We calculate the difference between the two phase values before and after propagation through the WSe_2_ monolayer and find a phase difference of 277 mrad because of the presence of the 4 μm wide WSe_2_ flake on the Au surface.

Given the wedge shape of the WSe_2_ crystal through which the SPP propagates, we can in a last step investigate its ability to act as a prism-like optical element on the surface inducing a variable dynamical shift in the SPP wavefront. To see this effect, we analyze the phase delay for all horizontal widths *w* of the monolayer material, which are plotted in Figure 4e. To this end, we determine the SPP oscillation phase for all positions in the image (see Figure S11 in the Supporting Information). Despite the width *w* being smaller than the SPP interference pattern wavelength, a clear dependence of the width of the flake is observed. To quantitatively determine the influence of the WSe_2_ prism part, we assume a proportional dependency of the phase shift Δφ on the material width *w*: Δφ = *c*·*w*. To avoid the uncertainty of determining the phase for a reference position, we only fit the slope c and normalize the absolute phase shift to zero using the fit (lines in Figure 4e). The fit to the red curve sets the starting conditions for the phase measurement on the left of the WSe_2_ crystal. Since the edge of the bulk WSe_2_ crystal resembles, in good approximation, a straight line, we fit a linear curve to the phase delays for different effective widths *w*. As expected from the almost perfect vertical alignment of the excitation edge, the phase change extracted from the slope is small: −4 mrad/μm. In stark contrast, we find a slope of 66 mrad/μm after the SPP has passed through the crystal shown in red in Figure 4e. By taking the difference, we find *c* = 70 mrad/μm, which agrees well with the previously determined value of 277 mrad for *L* = 4 μm.

Opposed to previous experiments that resolve phase delays by different dielectrics spatially, we performed a time-resolved experiment and hence express the measured delay also in the absolute experimental unit (attoseconds). We find a temporal phase delay of 30 as/μm (oscillation period of the SPP is 2.7 fs). Hence, a variation of the width of the WSe_2_ flake of 10 nm, assuming in a first approximation that the dielectric properties do not change much,^39^ would result in a shift of 0.3 as. Given that lithographic control at that level is available^40,41^ this shows that delaying the SPPs differently across the surface with a temporal precision down to 1 as is possible, opening up for on-surface spatiotemporal manipulation of the SPPs with TMDs as 2D optical elements. Since the 2D WSe_2_ has an integral atomic layer height, no height fluctuations through the flake will be present. Preparation of WSe_2_ flakes and combination with more advanced plasmonic devices under vacuum conditions and subsequent protection with an additional dielectric will further improve the potential device quality.

Calculations (see the SI) confirm the influence of the monolayer WSe_2_ crystal on the SPP delay. For a propagation distance of *L* = 4 μm, we numerically find a phase shift of 120 mrad. Normalizing it to the material width *w*, we get a shift *c*_*numerical*_ = 30 mrad/μm, which is on the same order of magnitude as the experimental data but approximately a factor of 2 smaller. That a discrepancy remains is not surprising: Due to its extremely short time duration, the laser pulse spans a significant spectral wavelength region in which the dielectric function varies significantly. The optical properties of the WSe_2_ and its exciton resonances in particular will depend on the specific sample conditions, which can be controlled, but are still different from the ones used as a basis for the calculations.^12^ Finally, the calculations are mean field approximations, and it is an open question if the atomic scale nature of mono- to few layer systems as well as quantum mechanical effects could set in at this point. While the present study demonstrates the concept of using 2D TMDs for temporal control and dynamics studies of atomic scale confined systems, it also indicates that further studies into the detailed temporal dynamics of SPPs in this 2D semiconductor–metal hybrid system would be interesting.

## Conclusions

In this work, we demonstrated, by broadband laser pulses, the efficient launching of SPPs from the edges of single and multilayer WSe_2_ which are much thinner than in the previously employed geometries for excitation. The SPP excitation dependence on the layer thickness and its orientation with respect to the light source can be well replicated from finite element modeling based on the dielectric properties. We observe complex interference patterns, which can be precisely predicted by geometric models of the waves using the crystal geometry. Thus, specific plasmonic field patterns can be designed by shaping of the TMDs based on simple modeling. Finally, we investigate attosecond temporal changes of the SPP varying in space as it propagates through a prism-like 2D WSe_2_ crystal. It can be concluded that high spatiotemporal SPP control is possible by technologically feasible manipulation of the 2D material. While the present work has been carried out on WSe_2_, many of the TMDs should display similar effects as they have values of the dielectric function in the same range.^12^ Additionally, launching and controlling SPPs by 2D TMD materials is easily realizable as the placement of such materials on flat metal films is a simple and widely available method—with samples prepared and transported in ambient air. A first example of this is the use of a prism-shaped WSe_2_ 2D crystal. The well-defined, crystalline nature of 2D TMDs lends itself well for a detailed fundamental study of SPPs by spatial and temporal imaging at the highest resolution. The observed intricate SPP interference patterns can be tailored for a number of uses, for example, collimated beams of SPPs,^42,43^ 2D plasmonic metasurfaces,^44^ SPP lenses,^45^ and nonlinear optics potentially combined with electrical gating to alter the pattern formation.^46^ It will be interesting to explore further hybrid material combinations of 2D dielectrics and metals as a playground for plasmonic control.

## Materials and Methods

### Sample

The smooth gold film was prepared by the template stripping method.^47^ Aluminum oxide (4.2 nm) was deposited onto the gold film by atomic layer deposition, and WSe_2_ flakes were mechanically exfoliated onto the prepared substrate. The aluminum oxide layers were utilized to eliminate the charge transfer processes between WSe_2_ and the gold film;^48^ however, it is not essential for the excitation of the SPPs. Samples are precharacterized using optical microscopy and atomic force microscopy to verify the WSe_2_ flake thicknesses (see the SI). By varying the laser power on a monolayer sample, we find a nonlinearity of the multiphoton electron emission process of 3.2 (see the SI), corresponding to three to four photons needed for different parts of the full laser spectrum. The same nonlinearity is observed at positions not covered by a WSe_2_ crystal.

### Laser/PEEM Experimental Setup

To evaluate the behavior of our plasmonic system, high spatial and temporal resolution is desirable. High temporal resolution is achieved by using few-cycle optical pulses from a Ti:Sa oscillator laser that are amplified in two consecutive noncollinear optical parametric amplifier stages to have a high pulse energy and repetition rate,^49^ while maintaining a pulse duration of ∼7 fs. A higher spectral resolution of the optical excitation of SPPs is achieved by employing an amplitude and phase pulse shaper and cutting spectral parts with a fwhm of 30 nm. The material dispersion due to the propagation of the pulses from the laser to the sample inside the vacuum chamber is compensated using chirped mirrors and a wedge pair.

To achieve an ∼40 nm resolution of the near field patterns induced by the light and SPPs, we exploit that the intensity of multiphoton emitted electrons from the surface will scale with the field to the power of 2*n* (*n* is the number of photons needed to excite over the work function threshold). As a result, the intensity of the collected photoelectrons corresponds to the combined field strength of the SPP and the exciting light on the surface. The light and SPP fields penetrate sufficiently deep into the surface to excite electrons in Au, Al_2_O_3_, and WSe_2_. The observed nonlinearity of the photoemission process (see above) indicates that electrons escape a (work function) barrier of ∼5.1 eV. This is significantly lower than the barrier for valence band electrons of Al_2_O_3_ to escape into a vacuum. However, it matches the work functions of Au and the barrier for valence electrons in WSe_2_ (electron affinity plus band gap) rather well,^50^ showing that electrons can be emitted from these two materials (see the SI for an excitation scheme). Since low-energy electrons can penetrate a monolayer of WSe_2_ as well as the Al_2_O_3_ layer and be imaged in PEEM,^51^ emission will most likely be coming from both Au and WSe_2_. The photoemission processes and transport to the surface in the first few nanometers are described in detail elsewhere,^52^ but for the present study the power of 6 electric field dependence of the PEEM intensity^53^ is the important point. The emitted electrons are collected by the extractor lens of a photoemission electron microscope (IS-PEEM, Focus GmbH, no energy filtering applied), and their spatial emission pattern is imaged onto a 2D detector with a spatial resolution down to ∼40 nm. We choose the exciting laser intensity such that in the high-field regions the field strength becomes strong enough to locally induce electron emission in a multiphoton photoemission process. By imaging the electron emission spots, the spatial distribution of the local fields is recorded.

### Finite Element Method Simulations

All numerical simulations in this study are performed for a light wavelength of 715 nm, and the corresponding optical constants are adopted from experimental measurements, with a permittivity of −17.560 + 1.1071i for Au,^54^ 3.107 for Al_2_O_3_,^55^ and 15.64 + 2.30i for WSe_2_,^12^ where the thickness of monolayer WSe_2_ is 0.65 nm. The simulations are performed in a wide region (>140 μm) with perfectly matched layer boundary conditions to include several fringe periods. The substrate is partially covered by a semi-infinite WSe_2_ crystal, with an edge at the origin (see Figure 2a). The incident Gaussian beam with a full waist of 16 μm and a maximum electric field amplitude of 1 V/nm is focused onto the WSe_2_ edge.

### Analytical 2D Pattern Simulation

The edge positions are extracted from the optical image shown in Figure 1. The wavelength of the SPPs is calculated using the dielectric constant of gold, and minor differences in the dielectric constant for propagation through WSe_2_ can be safely ignored in this context of qualitative image calculation. Further, the angle α between the in-plane *k*-vector of the laser field and the SPP propagation direction is determined using geometrical considerations. From this, the wavelength of the interference pattern *λ*_*I*_ can be calculated according to .^34^ However, here this calculation is carried out numerically by letting the SPP wave with field *E*_*SPP*_(φ) propagate from an edge (with laser field phase φ) and interfering it with the in-plane laser field *E*_*L*_(φ): *E*_*I*_i__ = *E*_*SPP*_(φ) + 3*E*_*L*_(φ). The ratio between the SPP and the laser field is not known, and a factor of 3 is estimated by comparison of the experimental and numerical fringe visibilities on the background. To further improve the agreement between the model and the experiment, we assume a decay length of the SPP of 12 μm, in agreement with calculations using the dielectric function of gold. Fields from different edges are coherently added, *E*_*tot*_ = ∑_*i*_*E*_*I*_i__, and this calculation is repeated for different phases φ. In the last step, the actual detected signal *I* is calculated using the nonlinearity *n* = 3 of the emission process: *I* = ∑*_φ_*|*E*_*tot*_(φ)|^2*n*^.

## Supporting Information Available

More information about the laser pulse characterization, a thickness evaluation of the WSe_2_ samples, electron emission dependence on laser pulse polarization, nonlinearity of the electron emission process, an excitation scheme, wavelength-dependent measurements, a numerical simulation of SPP propagation, and the electron autocorrelation data (PDF)

## Notes

The data that support the findings of this study are available from the corresponding authors on reasonable request.
