# Supplementary material for: Coherent Excitation and Control of Plasmons on Gold Using Two-Dimensional Transition Metal Dichalcogenides
Source: ACS Photonics. 2021 May 26;8(6):1607–15. doi: 10.1021/acsphotonics.0c01795 (PMC8296589; doi:10.1021/acsphotonics.0c01795)
Supplement: Supplementary file 1 — ph0c01795_si_001.pdf [file ph0c01795_si_001.pdf]

Supporting information for

Coherent excitation and control of plasmons on gold using two-dimensional transition metal dichalcogenides.

Jan Vogelsang, Lukas Wittenbecher, Deng Pan, Jiawei Sun, Sara Mikaelsson, Cord L. Arnold, Anne L'Huillier, Hongxing Xu, Anders Mikkelsen

Including 12 figures on 13 pages.

Laser pulse characterization

The spectrum of the laser pulses is shown in Fig. S1.

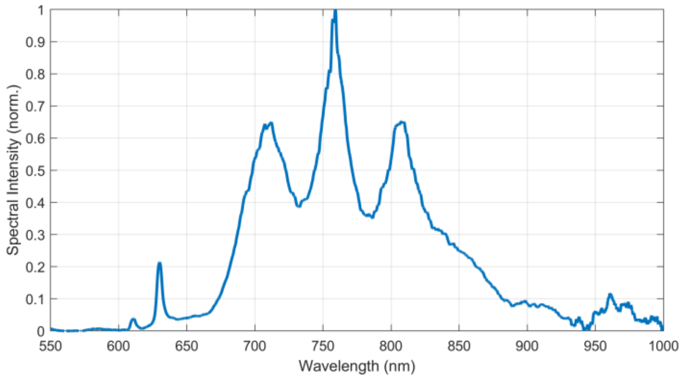

Figure S1. *Spectrum of the laser pulses supporting a pulse duration of less than 7 fs.*

The laser pulses used in the time-resolved part of the experiment were characterized using a frequency-resolved second harmonic autocorrelation measurement. Dispersive elements were used according to the actual beam path (chamber window, air). The electric field of the pulses is retrieved using a frequency-resolved optical gating algorithm and overlaid with the measurements (Fig. S2, left). The agreement is very good, confirming the retrieved pulse duration of <7 fs full width at half of the maximum. Additionally, instead of using a second harmonic generation process, the pulse duration was measured *in situ* using the 3-order electron emission process as a nonlinearity. The calculated signal using the previously retrieved field agrees well with the measurement (Fig. S2, right).

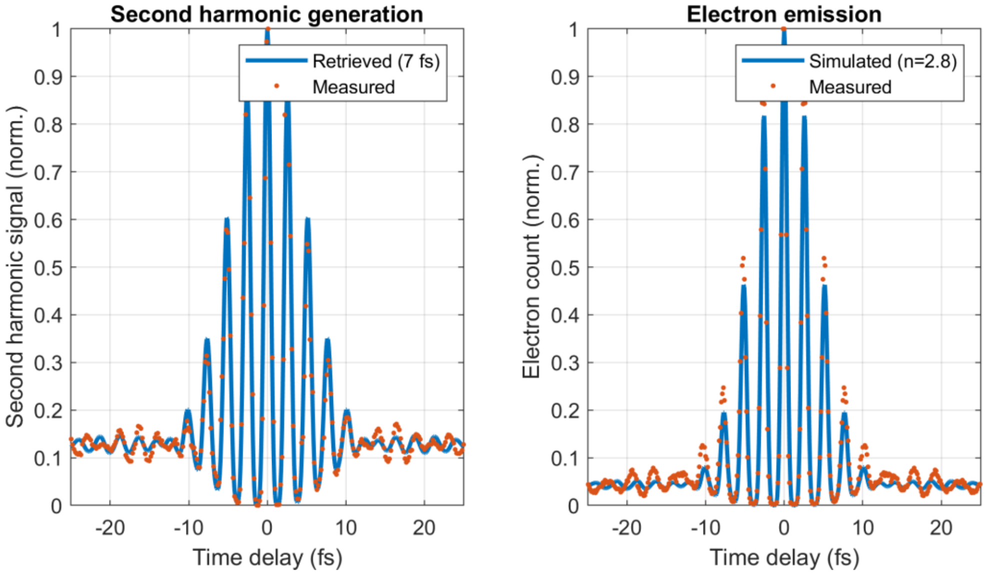

Figure S2. *Laser pulse characterization. Left: Autocorrelation measurement of the laser pulses recorded outside the vacuum chamber in a BBO crystal. The beam path length in air was comparable to the actual experiment and a separate vacuum chamber window was inserted into the beam path. Not only the second harmonic intensity but also the spectrum was recorded (not shown) and used to reconstruct the laser field using a standard frequency-resolved optical gating algorithm. A pulse duration of 7 fs is retrieved which matches the experimental data very well. Right: Experimental autocorrelation measurement performed inside the vacuum chamber using the nonlinear electron emission from bulk* WSe_2 *as the signal. Additionally, the expected signal is calculated using the electric field retrieved outside the vacuum chamber and a third order nonlinear emission process. The traces agree well.*

Thickness evaluation of WSe_2

Initially, the samples were selected using optical microscopy and the monolayer regions were identified by their specific color, well-known from previous measurements using photoluminescence and Raman spectroscopy[1]. The samples used in the experiments were additionally inspected using atomic force microscopy after the PEEM measurements and the result is shown in Fig. S3. The height of the monolayer region was evaluated by taking multi profiles in several places along the boundary of the WSe_2 flake. This was necessary due to the small height of a monolayer, thus even small variations in for example the underlying Al_2O_3 introduce noise. However, it could be confirmed that the part of the sample identified as 1 monolayer in the optical images had a height of less than 1 nm above the substrate, which agrees well with values found in literature for a single molecular layer.[2,3]

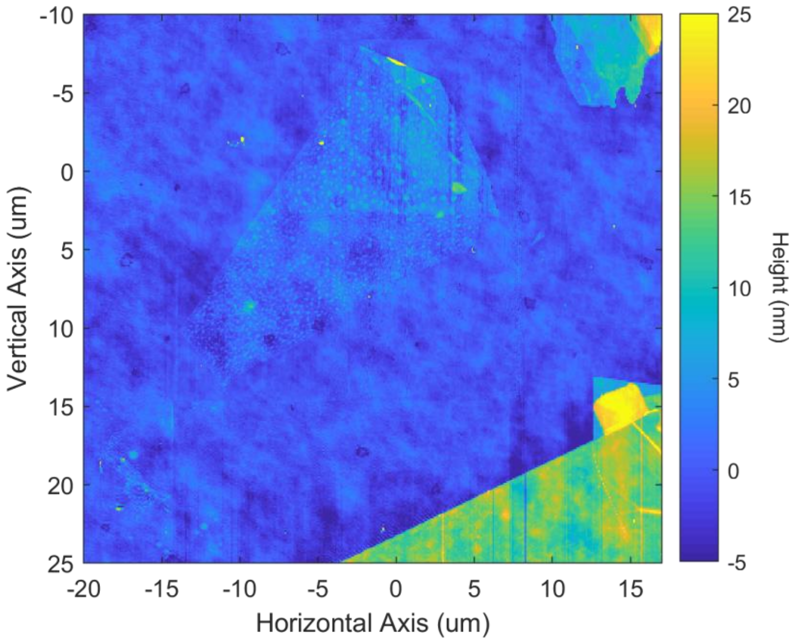

Figure S3. *Atomic force microscopy (AFM) height map of the first sample. The AFM measurements supports the conclusion from optical images that the central part of the sample consists of only a single layer of* WSe_2*.*

Electron emission dependence on laser pulse polarization

When the sample is illuminated with s-polarized instead of p-polarized light (as in the main manuscript), the electron emission signal is much weaker. Essentially, only emission from the bulk WSe_2 pieces is observed. This behavior was observed in other systems before and is expected due to a smaller transition moment for s-polarized illumination[4]. Interference effects are strongly suppressed. This is expected as the s-polarized laser field does not contain a field component normal to the surface. Hence, the modulation of the electron emission rate due to the normal laser field component interfering with the normal SPP field is reduced.

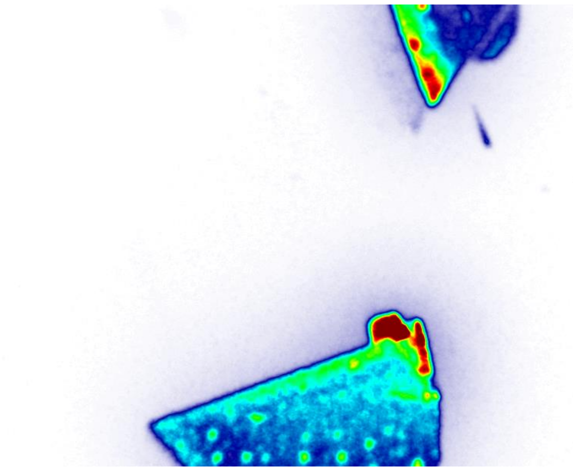

Figure S4. *PEEM image using s-polarized light. Electron emission from the same region of the sample as in Fig. 1 in the main text, but with s-polarized light. As expected*[3] *the SPPs are absent compared to p-polarized light.*

Nonlinearity of the electron emission process.

We adjust the optical power on the sample using two polarization filters and record the electron emission signal from a WSe_2 monolayer. Using the broad spectrum of our laser pulses, we find a nonlinearity of n=3.2. Due to the broad laser spectrum and hence broad energy range used for illumination, both 3- and 4-photon processes contribute to the emission signal. This is shown in Fig. S5.

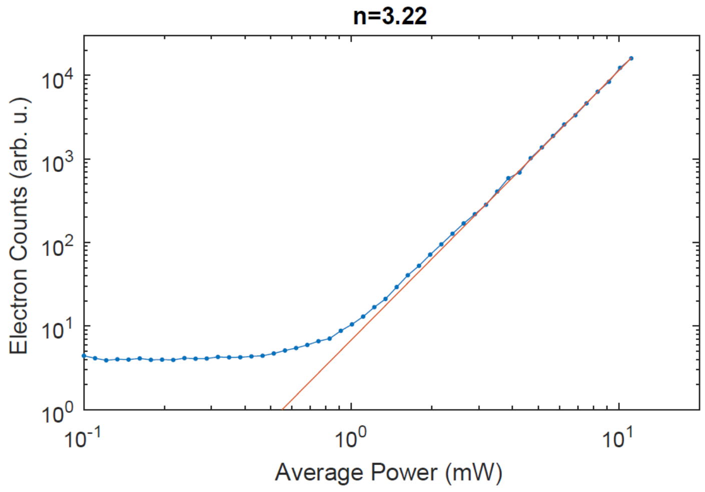

Figure S5. *Power-dependent measurement. Laser power dependent electron emission signal from the monolayer region shown in Figure 1 in the main text. By fitting the function* $y=c*x^n$ *to the data, a multiphoton order of n=3.2 is found. The broad optical spectrum explains the non-integer photon number.*

Excitation scheme

Two energy level diagrams including energy levels relevant for the photoemission process are shown in Fig. S6[5-8]. Different emission pathways with different numbers of photons (three to four) from different parts of the laser spectrum can contribute. Sequential emission via the Al_2O_3 conduction band is an option, but the expected long lifetime of those states is not observed in the time-resolved measurements. A more detailed discussion on the photoemission processes can be found elsewhere[9].

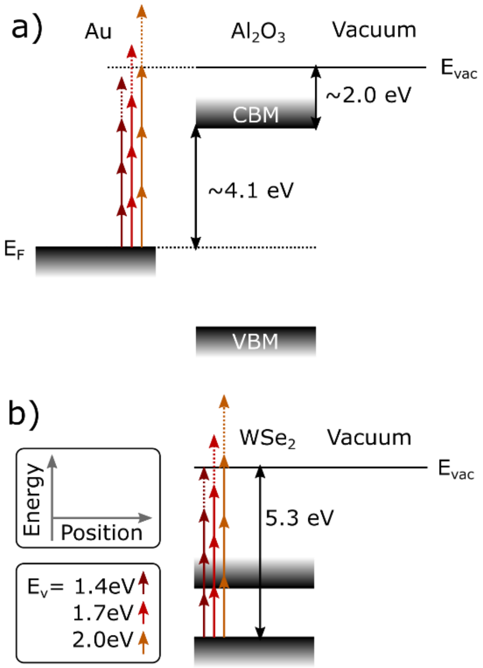

Figure S6. *Electron excitation scheme. a) Electron emission from gold via three to four photon internal photoemission with different photon energies (see b) followed by propagation through the Al_2O_3 layer. The values of barriers will depend on the precise surface, interface and material structure. b) Direct photoemission from WSe_2 via absorption of three to four photons.*

Wavelength-dependent measurements

To confirm the plasmonic nature of the interference fringes observed in the PEEM measurements shown in Fig. 1 in the main text, we performed wavelength-dependent

measurements of the interference pattern. This is shown in Fig. S7. In particular, in Fig. S7d-f, the change in fringe spacing for different wavelengths is clearly visible.

A rather subtle difference is the overall electron emission rate, but also SPP excitation efficiency from the WSe_2 flake for the different wavelengths. While for wavelengths of 715 nm and 745 nm the outlines of the monolayer are clearly visible due to an apparent enhancement of the electron emission rate from this monolayer region, this seems to be less prominent for the longest wavelength of 775 nm shown in Fig. S7c.

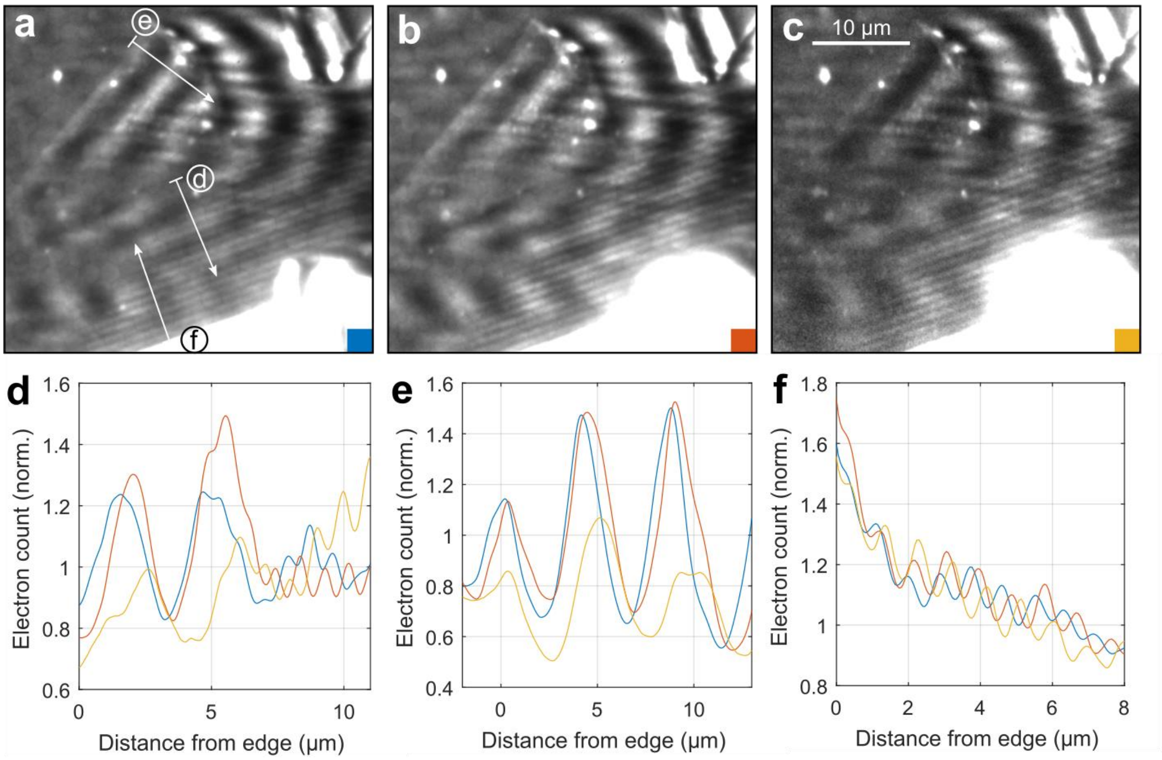

Figure S7. *SPP excitation for varying laser wavelengths.* ***a-c****, Photoemission electron microscope images with changing central laser wavelengths of 715 nm (see Fig. 1), 745 nm and 775 nm respectively. A change of the interference pattern corresponding to the change in laser wavelength can be observed. For the longest wavelength, the signal from the* WSe_2 *mono- and few-layer region is weaker.* ***d-f****, Lineouts along the white arrows shown in* ***a****. The color of the lines corresponds to the color marked in the bottom right corner of each image. Besides the wavelength change of the interference fringes, also here a change in signal strength for the lineouts belonging to the image shown in* ***c*** *is visible.*

This can be understood by considering the lowest energy exciton state A with an energy of 1.65 eV which corresponds to a photon wavelength of 751 nm. We cannot excite this exciton by absorption of a single photon at a wavelength of 775 nm anymore. This reduces the overall absorption and scattering cross section and hence the observed electron emission rate from the WSe_2 region as well as the excitation efficiency of the SPP. This is confirmed by comparing the lineouts: Apart from the change in periodicity, also the excitation efficiency clearly changes in the long-wavelength case.

Numerical simulation of SPP propagation

For SPP propagation over a distance d, the phase delay $k_{eff}d$ is determined by the SPP wave vector $k_{eff} = n_{eff}k_0$, where n_{eff} is the refractive index, as labelled in Fig. S8, and $k_0 = 2\pi/\lambda$ is the vacuum wave vector. The phase shift relative to $n_0 k_0 d$, the delay induced by the protected gold itself, is shown in Fig. S8. It demonstrates that a single atomic layer is enough to induce a considerable phase shift on the SPP propagation.

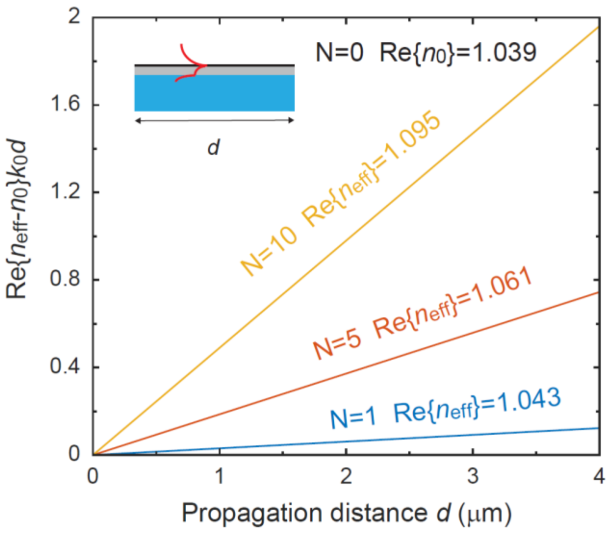

Figure S8. *Phase shifts. Calculated phase shift for SPP propagation through different number of layers N of WSe_2. Propagation on gold with a thin protection layer is taken as a reference as in the experiment. A phase shift of 120 mrad or 50 as is found after propagation of 4 µm for N=1.*

Electron autocorrelation data

In Fig. S9 the complete autocorrelation data used in Fig. 4 in the main text are shown.

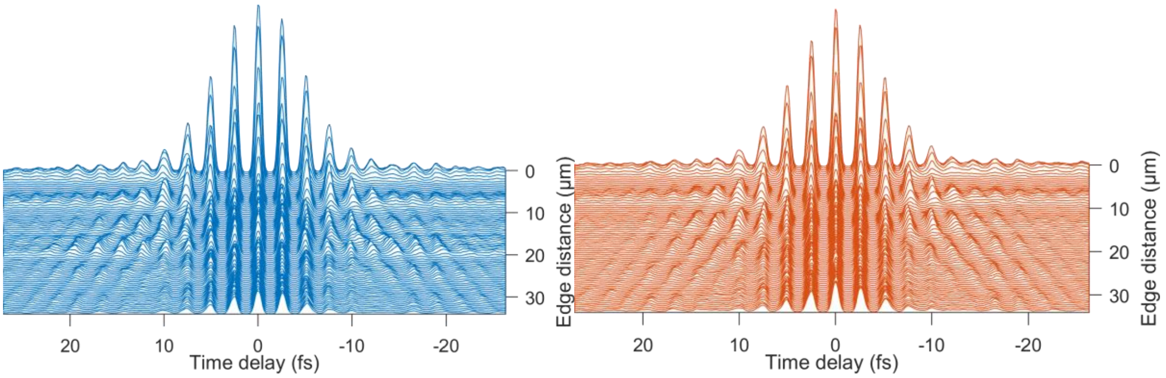

Figure S9. *Electron autocorrelation. The data for the two lines marked in Fig. 4c are shown. They are shifted vertically for clarity, labelled by their respective distance to the bulk WSe_2 edge on the left in Fig. 4c. The upper line is shown on the left, the lower one on the right.*

Determining the phase shift

The analysis shown in the main text in Figure 4d for four points marked with red and yellow crosses was repeated for neighboring positions to improve the statistics. The phase of the plasmon oscillation in the autocorrelation measurement was determined for a series of vertically aligned points using a Fourier transform and the phase difference was extracted. This was done both before and after part of the SPP has propagated through the WSe_2 monolayer.

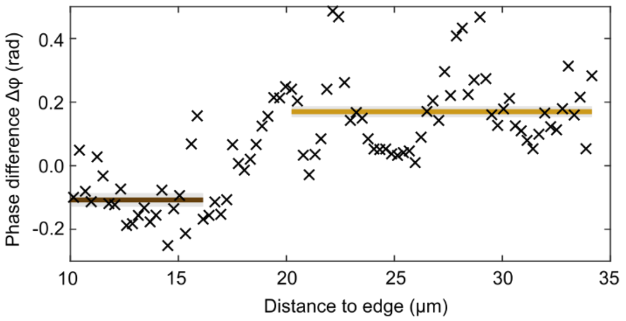

Figure S10. *Phase difference. The phase difference is determined for positions before and after the monolayer WSe_2 (see also Figure 4 in the main text). The width of the lines*

indicates the standard error of the mean. The region from 16.5 µm to 20.5 µm is not used for fitting, because it is the part where the monolayer is lying.

Fig. S10 shows that there is a clear negative phase difference close to the excitation edge, i.e. the SPP propagating towards the monolayer is ahead by 107 mrad due to a kink in the excitation edge. This turns around after part of the SPP has propagated through the monolayer. Now, the phase difference has an opposite sign and becomes +170 mrad. After having established that there is a clear phase difference before and after the monolayer piece, the shift is extracted for all widths of the monolayer L. For this, the phase of the SPP oscillation if extracted for all positions in the 2D PEEM images using a Fourier transformation. The resulting phase image is shown in Fig S11.

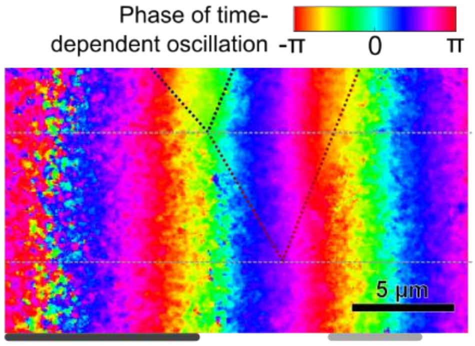

Figure S11. *Spatially resolved SPP phase. From the spatial images recorded for different time delays, the SPP oscillation phase at time delays larger than 10 fs is extracted and shown here. The blue and red dotted lines indicate the mono- and bilayer position. The horizontal dotted lines indicate the vertical region used for the analysis. The thick lines on the bottom indicate the left and right horizontal regions used.*

The image nicely visualizes how the interference pattern and with it the SPP propagates from left to right. For the vertical region marked by the dotted lines, a complex exponential function with a fixed spatial frequency and variable phase is fitted to the data in each horizontal line, both before and after the monolayer. Thus, the phase of the oscillation phase is extracted in the left region (marked with a dark grey bar below the image in Fig. S11) and

the right region (light grey bar). The difference between the resulting phase values is shown in the main text in Figure 4e. The data, having an arbitrary phase offset, is fitted using a linear function. The constant part of the fitting result is used to determine the absolute phase change and set it to zero for a material length L=0.

SPP excitation efficiency from a step

We compare the excitation efficiency of SPPs using a monolayer of WSe_2 to a step in Al_2O_3 with the same height. The simulation result is shown in Figure S12.

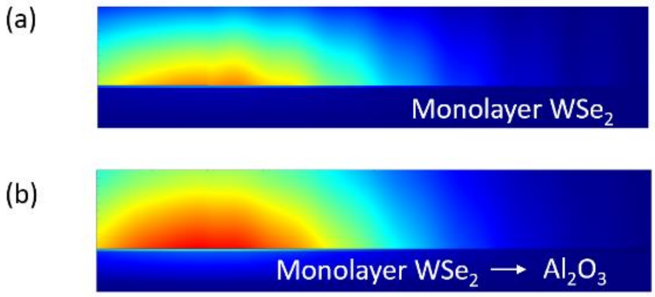

Figure S12. *Simulation of electric fields after optical excitation.* ***a****, Identical simulation to the one shown in Fig. 2 in the main text assuming a monolayer of WSe_2 placed on an Al_2O_3-covered Au surface.* ***b****, The dielectric function of WSe_2 is replaced by Al_2O_3, resulting in a small step relative to the underlying Al_2O_3.*

We find a clear reduction of the SPP excitation efficiency by comparing the modulation of the electric fields. While Fig. S12a shows the case of WSe_2 with a modulation of the laser focus due to interference with the SPP electric field, the modulation is almost completely absent in Fig. S12b.

References

(1) Sun, J.; Hu, H.; Zheng, D.; Zhang, D.; Deng, Q.; Zhang, S.; Xu, H. Light-Emitting Plexciton: Exploiting Plasmon–Exciton Interaction in the Intermediate Coupling Regime. *ACS Nano* **2018**, *12* (10), 10393–10402.

(2) Sahin, H.; Tongay, S.; Horzum, S.; Fan, W.; Zhou, J.; Li, J.; Wu, J.; Peeters, F. M. Anomalous Raman Spectra and Thickness-Dependent Electronic Properties of WSe 2. *Phys. Rev. B* **2013**, *87* (16), 165409.

(3) Zhou, H.; Wang, C.; Shaw, J. C.; Cheng, R.; Chen, Y.; Huang, X.; Liu, Y.; Weiss, N. O.; Lin, Z.; Huang, Y.; Duan, X. Large Area Growth and Electrical Properties of P-Type WSe_2 Atomic Layers. *Nano Lett.* **2015**, *15* (1), 709–713.

(4) Petek, H.; Ogawa, S. Femtosecond Time-Resolved Two-Photon Photoemission Studies of Electron Dynamics in Metals. *Progress in Surface Science* **1997**, *56* (4), 239–310.

(5) Yeo, Y.-C.; King, T.-J.; Hu, C. Metal-Dielectric Band Alignment and Its Implications for Metal Gate Complementary Metal-Oxide-Semiconductor Technology. *Journal of Applied Physics* **2002**, *92* (12), 7266–7271.

(6) Kamimura, T.; Sasaki, K.; Hoi Wong, M.; Krishnamurthy, D.; Kuramata, A.; Masui, T.; Yamakoshi, S.; Higashiwaki, M. Band Alignment and Electrical Properties of Al2O3/β-Ga2O3 Heterojunctions. *Appl. Phys. Lett.* **2014**, *104* (19), 192104.

(7) Xiao, J.; Zhang, Y.; Chen, H.; Xu, N.; Deng, S. Enhanced Performance of a Monolayer MoS2/WSe2 Heterojunction as a Photoelectrochemical Cathode. *Nano-Micro Lett.* **2018**, *10* (4), 60.

(8) Nikolaou, N.; Dimitrakis, P.; Normand, P.; Skarlatos, D.; Giannakopoulos, K.; Mergia, K.; Ioannou-Sougleridis, V.; Kukli, K.; Niinistö, J.; Mizohata, K.; Ritala, M.; Leskelä, M. Inert Ambient Annealing Effect on MANOS Capacitor Memory Characteristics. *Nanotechnology* **2015**, *26* (13), 134004.

(9) Brillson, L. J. Surfaces and Interfaces of Electronic Materials; John Wiley & Sons, 2010.
